# Study of Single Nucleotide Polymorphisms in Endosomal Toll-Like Receptors-3, 7, and 9 Genes in Patients With Dengue: A Case-Control Study

**DOI:** 10.7759/cureus.14883

**Published:** 2021-05-07

**Authors:** Arvind K Singh, Shantanu Prakash, Ravindra Kumar Garg, Parul Jain, Rashmi Kumar, Amita Jain

**Affiliations:** 1 Department of Microbiology, King George's Medical University, Lucknow, IND; 2 Department of Neurology, King George's Medical University, Lucknow, IND; 3 Department of Pediatrics, King George's Medical University, Lucknow, IND

**Keywords:** dengue, tlr 9, innate immunity, toll-like receptors, tlr3, tlr7

## Abstract

Introduction

The endosomal toll-like receptors (TLR) TLR3, TLR7, and TLR9 are localized in immune cells, recognize the viral pattern associated molecular pattern (PAMPs), and start signaling cascades for immune defense response and genetic factor is known to affect the dengue virus infection therefore in our study we study the association of endosomal Toll-like receptors 3 polymorphism rs3775291, rs3775290, and rs3775296 with rs179008 A/T and rs179009C/T polymorphisms of TLR7 and rs187084 (C/T), rs5743836 (C/T) of TLR9 gene with dengue and controls.

Materials and methods

Ninety-eight cases of dengue virus (DV) infection and 98 healthy controls were enrolled. Clinical details were recorded and cases were classified as severe or non-severe dengue, based on WHO 2009 classification. Genotypes were determined by Sanger sequencing using the genetic analyzer.

Results

An increased risk of DV infection was observed in cases as compared to controls, with TLR 3 rs3775291 CT genotype (OR = 4.34, P-value: 0.031, CI = 1.14-16.5), Likewise in TLR7 rs179008 AT (OR = 2.12, P-value: 0.034, CI = 1.06-4.26) and rs179009 CT (OR = 2.04, P-value: 0.040, CI = 1.03-4.05) same as in TLR9 rs187084 CT (OR = 1.97, P-value: 0.046, CI = 1.013-3.84) and rs5743836 (OR = 2.38, P-value: 0.009, CI = 1.24-5.57). In the above polymorphisms, mutant allele was observed in a significantly higher number in cases. The values are: for TLR3 rs3775291 T allele (OR 2.167, CI = 1.3-3.61, P-value: 0.003). TLR7 rs179008 T allele (OR 1.90, P-value: 0.021, CI = 1.07-3.35) and in TLR9 rs187084 (OR 1.91, P-value: 0.014, CI = 1.137-3.24) rs5743836 (OR 2.29, P-value: 0.0018, CI = 1.36-3.87). No significant association was observed in the genotypic frequency of severe versus non-severe dengue.

Conclusion

TLR3, 7, and 9 gene polymorphism might confer host genetic susceptibility to dengue in the North Indian population

## Introduction

Dengue has become a global health problem as it frequently causes epidemics throughout the tropical, subtropical, and temperate regions of the world [1]. World Health Organization (WHO) estimates a 30-fold increase in incidence in the last 50 years. Approximately 100 million cases of dengue fever, over 500,000 cases of dengue hemorrhagic fever, and 25,000 fatal cases occur annually [2]. The complexity of variable clinical outcomes, ranging from self-limiting asymptomatic infection to severe dengue and death, has been attributed partially to the host genetic factors as recognition of pathogens through the pattern recognition receptors (PRRs) is the first step in the initiation of immune responses. Toll-like receptors (TLRs) are one of the categories of PRRs that are present in abundance on immune cells like monocytes, macrophages, and dendritic cells, which are instrumental in regulating host immune responses against viruses.

TLR3, 7, and 9 are expressed on endosomes of dendritic cells (DCS), epithelial cells lining the airway, genital tract, biliary tract, intestine, and neurons. On binding with RNA of the infecting virus, they run a cascade of reaction by initiating the activation of natural factors (NF-kB) and interferon regulatory factor (IRF-3), which in turn regulates expression of type I interferon (IFNs) and thus contribute to antiviral response [3]. Single nucleotide polymorphisms (SNPs) in these TLR regions have been shown to be associated with outcomes of various viral infections such as hepatitis C, cytomegalovirus, HIV, etc. as well as nonviral diseases like cancers and autoimmune diseases. Few SNPs have been shown to affect the functions of TLRs as a result of amino acid changes [4-7].

Therefore, given the complex pathogenesis of dengue fever, the present study was carried out to decipher the associations of polymorphisms in TLR3, 7, and 9 genes with susceptibility and severity of dengue illness.

## Materials and methods

Study design

This prospective case-control study was done in Departments of Microbiology, Neurology, and Pediatrics at King George’s Medical University, at a tertiary level health care center in Lucknow, Uttar Pradesh. Ethical approval was obtained from the institutional ethics committee (ECR/262/Inst/UP/2013). After obtaining written informed consent, cases and controls were enrolled in geographical regions and those who were laboratory-confirmed negative for dengue virus infection were enrolled as controls. For assessment of dengue virus infection patients’ serum was tested by ELISA for anti-dengue IgM (NIV, Pune, India) and non-structural-1 antigen (NS1Ag) (J. Mitra and Company, India) using manufacturer’s instructions. The sample size was calculated by QUANTO software; values used were: odds ratio (OR): 2, significance level (α): 0.05, Power of study: 90%, minor allele frequency: 0.2 (using db SNP), disease frequency: 18%. Clinical details were recorded in a pre-designed questionnaire and routine blood investigations including complete blood count, liver and renal function test, and electrolytes were measured in all patients. Cases were classified as severe or nonsevere dengue, based on WHO 2009 classification [8]. Analysis of TLR3 polymorphism: Blood samples (3 ml) collected from enrolled cases and controls were subjected to the salting-out method [9] for extracting genomic DNA, which was stored at −20°C until analyzed. TLR3, 7, and 9 genotypes were determined by polymerase chain reaction-sequencing by chain termination method (PCR-sequencing). Primers for sequencing were designed in-house and manufactured by Integrated DNA technology, USA. Sequences used are given in Table 1 along with their product sizes.

**Table 1:** Primer sequences and amplification products for TLR3, TLR7 and TLR 9 polymorphisms. TLR: toll-like receptor.

Gene	Polymorphism	Primer sequence	Product size
TLR3	rs3775290 and rs3775291	Forward 5′-TCACAGGATTGATAAACCTGAAAT-3′; reverse 5′-AAAGACAGATGCTGGAAGTGTG-3′	550bp
TLR3	rs3775296	Forward 5′-GCAACTGGCATTAAGGTGAA-3′; reverse 5′-GGGAAGTTTCTGGCACAATTC-3′	510bp
TLR7	rs179008, rs179009	Forward 5′-TGGCAGTGAATCTATGGCTCT-3′; reverse 5′-CCAGGAATTTCTGTCAAATGC-3′	493bp
TLR9	rs187084, rs5743836	Forward 5′-TCATTCAGCCTTCACTCAGAAA-3′; reverse 5′-TCAAAGCCACAGTCCACAGA-3′	557bp

All PCR amplifications were performed in a 25-µL volume of master mix, containing 10X assay buffer, 200 µM each of nucleotide, 1 pm of each primer, 1.0 U of Taq DNA polymerase (Finzymes, Thermo scientific). PCR conditions were as follows: an initial denaturation at 94°C for 10 min, followed by 35 cycles of denaturing at 94°C for 45 sec, annealing at TLR3 (51.6°C), TLR7(55.2°C), TLR9(55.5°C ) for 45 sec, extension at 72°C for 1 min, final extension at 72°C for 7 min and cooling to 4°C. Template-free water was used as a negative control. After amplification, the products were subjected to exo-sap purification and the purified 1-2 µL PCR products were subjected to bidirectional sequencing PCR utilizing BigDye Terminator cycle sequencing Kit v3.1 (Thermo Fischer Scientific, USA) and single primer. The amplified sequencing PCR products were again purified by ethanol, EDTA, and sodium acetate precipitation. SNPs were analyzed by software BioEdit 7.2.1 sequence alignment editor [10]. The genotypes and the alleles of studied TLR3 polymorphisms were compared between cases and controls for determining the significant association of TLR3 with the susceptibility to dengue.

Statistical analysis

Statistical analysis was done using SPSS version 16.0 (Chicago, IL). All categorical variables were expressed as percentages and continuous variables were expressed as mean ± standard deviation. Chi-square test or Fisher exact test was used to compare categorical variables as applicable. Independent sample T-test and Mann-Whitney test was used to compare means. Binary logistic regression was performed to look for variables independently associated with outcome. We used the SNP analyzer version 2.0 web-based programme20, to determine deviation from Hardy-Weinberg equilibrium (HWE), linkage disequilibrium, association analysis and to determine haplotype frequencies. The most frequent homozygous genotype in the control group was used as a reference for association analysis. Statistical significance was determined at P<0.05 for all tests odds ratios (ORs) for this analysis with the corresponding 95% confidence intervals (95% CIs) were computed.

## Results

Demographic features of the subjects

A total of 98 cases and controls were enrolled in the study. The minimum and maximum age of cases were 5 and 62 years, respectively (mean age: 37.7) and of controls were 7 and 71 years, respectively (mean age: 35.98). Of the total 98 enrolled dengue cases, 84 presented as dengue fever, and 14 presented as severe dengue. The most common symptoms were vomiting, headache, body ache, and arthralgia. Table 2 details the demographic and clinical data of cases and control.

**Table 2:** Demographic and baseline clinical and laboratory characteristics of cases. Chi-square test or Fisher exact used to compare the mean. TLC: total leucocyte count; SGPT: serum glutamic pyruvic transaminase; SGOT: serum glutamic oxaloacetic transaminase; ALP: alkaline phosphatase.

SN	Characteristics		Case, N=98 (%)	Control, N=98 (%)	P-value
Demographic characteristics					
1.	Age (years): mean±SD		37.70±0.81	35.98±0.77	0.975
2	Sex:				
	Female		62(63)	59(60)	0.76
	Male		36 (36)	39 (39)	0.77
Clinical features			Dengue (N=84) (mean±SD)	Severe dengue (N=14) (mean ±SD)	P-value
3.	Vomiting		31 (36.90%)	5 (28.57%)	0.932
4	Headache		12 (36%)	5 (%)	0.128
5.	Bodyache		33 (39.28%)	10 (71.42%)	0.050
6.	Abdominal pain		24 (32.14%)	6 (42.85%)	0.479
7.	Weakness		12 (14.28%)	2 (14.28%)	>0.999
8.	Altered behaviour		0 (0%)	5 (35.71%)	<0.001
9.	Seizures		0 (0%)	3 (21.42%)	<0.001
10.	Bleeding		0 (%)	14 (%)	<0.001
11.	Hematocrit		36.07±7.9	41.22±4.2	0.020
12.	Platelet count(10^3^/mm^3^)		0.57±0.5	0.18±0.06	0.003
13.	Haemoglobin		12.38±2.5	11.93±2.77	0.251
14.	TLC		7667.58±4798	4851±2223	0.125
15.	Na		135.44±15	139.03±4.48	0.300
16.	K		4.2±0.819	4.0±0.501	0.332
17.	Creatininne		1.09±0.60	1.19±0.44	0.493
18.	Urea		40.30±22.34	40.95±23.44	0.738
19.	T Bill		1.32±1.83	0.79±0.25	0.128
20.	SGPT		128±161.8	181±164	0.030
21.	SGOT		200±376	206±151	0.120
22.	ALP		288±284	273±182	0.772
23.	Protein		6.10±1.22	6.33±0.89	0.468
24.	Albumin		3.39±0.66	3.76±0.411	0.348

Genotypic and allelic distribution

Table 3 summarizes findings on the allelic and genotypic distributions of TLR3, TLR7, and TLR 9 gene SNPs in all the cases and controls.

**Table 3:** TLR3, TLR7, and TLR9 genotype frequency distribution of gene polymorphism. Binary logistic regression used for genotype frequency.

Polymorphism	Dengue case (N=98)	Control (N=98)	Non-severe dengue (N=84)		Severe dengue (N=14)	Case vs. control						Non-severe vs. control			
						P-value			OR		(CI)	P-value		OR	(CI)
TLR3 rs3775290															
Homozygous CC	58(59.2%)	70(71.5%)	52(61.9%)		6(42.9%)	1						1			
Heterozygous CT	34(34.7%)	23(23.46%)	27(32.1%)		7(50%)	0.557			1.448		(0.420-4.989)	0.181		2.247	0.69-7.35
Mutant TT	6 (6.1%)	5(5.10%)	5(6%)		1(7%)	0.753			0.812		(0.221-2.977)	0.640		1.733	0.17-17.41
C allele	150(76.5%)	163(83%)	131(77.9%)		19(67.8%)	1						1			
T allele	46 (23.4%)	33(16.83%)	37(22.02%)		9(32%)	0.10			1.51		(0.91-2.49)	0.24		1.67	0.70-4.01
TLR3 rs775291															
Homozygous CC	56(57%)	73(74.5%)	51(67%)		5(35%)	1						1			
Heterozygous CT	32(32.7%)	22(22.4%)	26(31%)		6(42.9%)	0.05			1.89		(0.99-3.61)	0.186		2.45	0.65-8.44
Mutant TT	10(10.2%)	3(3.1%)	7(8.3%)		2(21.4%)	0.031			4.34		(1.14-16.5)	0.77		4.371	0.85-22.42
C allele	144(73.4%)	168(85.7%)	128(76.1%)		16(5%)	1						1			
T allele	52(26.5%)	28(14.2%)	37(22.02%)		6(42.8%)	0.003			2.16		(1.3-3.61)	0.038		2.4	1.04-5.40
TLR3 rs3775296															
Homozygous CC	63(64.3%)	76(77.55%)		57(67.9%)	6(42%)	1						1			
Heterozygous CA	28(28.6%)	19(19.38%)		22(26.2%)	6(42%)	0.145			2.815		(0.088-1.431)	0.130		2.591	0.75-8.80
Mutant AA	7(7.1%)	3(3.1%)		5(6%)	2(14.3%)	0.541			1.583		(0.145-2.75)	0.56		3.80	0.60-23.99
C allele	154(78.5%)	171(87.2%)		136(80.9%)	18(64.2%)	1						1			
A allele	42(21.4%)	29(15.7%)		32(19.04%)	10(35.7%)	0.07			1.608		(0.95-2.70)	0.051		2.36	0.99-5.6
TLR7 rs179008 A/T															
Homozygous AA	65(66.33%)	79(80.61%)		11(78.57%)	54(64.28%)		1						1		
Heterozygous AT	28(28.57%)	16(16.32%)		03(21.42%)	25(29.76)		0.034	2.12		(1.06-4.26)			0.78	1.22	0.29-5.02
Mutant TT	5(5.10%)	3(3.06%)		00(0%)	05(5.9%)		0.346	2.02		(0.466-8.79)			0.95	1.06	0.10-10.50
A allele	158(80.61%)	174(88.77%)		25(89%)	133(79.1%)		1						1		
T allele	38(19.28%)	22(11.22%)		3(10.7%)	35(20.8%)		0.021	1.90		(1.07-3.35)			0.24	0.47	0.134-1.65
TLR7 rs179009 C/T															
Homozygous CC	65(66.32%)	78(79.59%)		11(78.57%)	54(64.2%)		1						1		
Heterozygous CT	29(29.59%)	17(17.34%)		03(21.42%)	26(30.9%)		0.040	2.04		(1.03-4.05)			0.81	0.75	0.075-7.60
Mutant TT	4(4.08%)	3(3.06%)		0(0%)	4(4.76%)		0.548	1.60		(0.34-7.40)			0.523	0.42	0.032-5.77
C Allele	159(162.2%)	173(88.26%)		25(89.2%)	134(79.7%)		1						1		
T Allele	37(37.75%)	23(11.3%)		03(10.7%)	34(20.2%)		0.051	1.75		0.99-3.07			0.24	0.47	0.134-1.65
TLR9 rs187084 (C/T)															
Homozygous CC	60(61.22%)	75(76.5%)		54(64.28%)	7(50%)		1						1		
Heterozygous CT	30(30.61%)	19(19.38%)		23(27.38%)	6(42.85%)		0.046	1.974		1.013-3.84			0.45	1.60	0.464-5.532
Mutant TT	8(8.16%)	4(4.08%)		7(8.33%)	1(7.14%)		0.150	2.50		0.718-8.702			0.83	1.27	0.124-12.617
C Allele	150(76.5%)	169(86.2%)		131(77.9%)	20(71.41%)		1						1		
T Allele	46(23.46%)	27(13.77%)		37(22.02%)	8(28.5%)		0.014	1.91		1.137-3.24			0.44	1.41	0.577-3.47
TLR9 rs5743836 (C/T)															
Homozygous CC	55(56.12%)	75(76.53%)		48(57.14%)	7(50%)		1						1		
Heterozygous CT	35(35.71%)	20(20.40%)		28(33.33%)	7(50%)		0.009	2.38		1.24-5.57			0.48	1.53	0.468-5.033
Mutant TT	8(8.1%)	3(3.06%)		8(9.52%)	0(0%)		0.065	3.636		0.922-14.33			0.999	NA	NA
C Allele	145(73.9%)	170(86.7%)		124(73.8%)	21(75%)		1						1		
T Allele	51(26.02%)	26(13.26%)		44(52.38%)	7(25%)		0.0018	2.29		1.364-3.874			0.89	0.93	0.37-2.36

TLR gene polymorphism

On genotypic analysis of TLR3 rs3775291 polymorphism, heterozygous genotype (CT) was more common in cases compared to controls, (32.7% vs. 22.4%, OR = 1.89, P-value = 0.050, CI = 0.995-3.614,), though the difference was not statistically significant. Statistically significant mutant genotype (TT) (110.2% vs 3.1% OR= 4.34, P-value = 0.031, CI = 1.14-16.5) was observed more in cases than controls. The difference in mutant (T) allele frequency in cases than control was significant (26.5% vs. 14.2% OR = 2.167, P-value = 0.003, CI = 1.3-3.61) (Table 3).

On genotype analysis, rs3775290 heterozygous genotype (CT) was found more frequently in cases than controls but difference was not significant (34.7% vs. 23.46%, P-value = 1.448, CI = 0.420-4.989). Mutant allele was found more in cases compared to controls (23.4% vs. 16.83%, OR = 1.51, P-value = 0.10, CI = 0.91-2.49) but difference was not significant (Table 3).

 Higher frequency of heterozygous genotype (CA) of TLR3 rs3775296 polymorphism was seen in cases as compared to control (28.6% vs. 19.3%, OR = 2.815, P-value = 0.145,CI = 0.088-1.431). No statistical significance was found in cases having mutant genotype (AA) compared to controls (7.1% vs. 3.1%, OR = 1.431, CI = 0.145-2.75, P-value = 1.583). The frequency of mutant (A) allele in case and control was high (21.42% vs. 15.7% OR = 1.608, CI = 0.95-2.70, P-value = <0.07) (Table 3).

TLR 7 gene polymorphism: On genotypic analysis of TLR7 rs79008 A/T polymorphism frequency, heterozygous genotype (AT) was more common in cases compared to controls, (28.57% vs. 21.42%, OR = 2.12, P-value = 0.034, CI = 0.29-5.02). The difference in mutant (T) allele frequency in cases with control was significant (19.28% vs. 11.2% OR = 1.90, P-value = 0.021, CI = 1.07-3.35) (Table 3).

On genotype analysis of TLR7 rs179009C/T polymorphism, heterozygous genotype (CT) was found significantly high in cases compared to controls (29.59% vs. 17.34% OR = 2.04, P-value = 0.040, CI = 1.03-4.05) (Table 3).

TLR 9 gene polymorphism: On genotype analysis of TLR9 rs187084C/T polymorphism, heterozygous (CT) genotype was significantly high (30.61% vs. 19.38%, OR = 1.97, P-value = 0.046, CI = 1.013-3.84) in cases than controls.

On TLR9 rs5743835 (C/T) polymorphism heterozygous (CT) genotype was more common in cases than control (35.71% vs. 20.40%, OR = 2.38, P-value = 0.009, CI = 1.24-5.57) and this difference was significant. Likewise mutant allele was also found significantly high in cases (26.02% vs. 13.26% OR = 2.29, P-value = 0.0018, CI = 1.36-3.84) (Table 3).

No significant association was observed in the genotypic frequency of severe versus nonsevere dengue (Table 3).

## Discussion

Dengue infection ranges in severity from mild disease with headache and fever to a severe disease with plasma leakage or shock, which may even lead to death (WHO). The clinical condition is partially attributed to the host’s genetic composition required for viral clearance. TLRs play a pivotal role in the activation of innate immunity against viral infection. TLR3 is sensitized by ds RNA that further starts a cascade of chain reaction via the production of several chemokines and cytokines. In the present study, we observed an association between TLR3 rs3775291 (Leu412ple) and rs3775290 polymorphisms and dengue virus infection. The T allele of TLR3 rs3775291 and A allele of rs3775296 were more frequently seen in cases as compared to controls in the north Indian population. The s3775291 polymorphic site is present on the 412th codon and a mutational change can alter the amino acid from leucine to phenylalanine. Thus, mutant ‘T’ allele codes for phenylalanine in rs3775291 which has been shown to play a crucial role in the genetics of diseases like subacute sclerosing panencephalitis [11]. A study conducted by Liang Z et al. 2011 revealed that the activation of TLR3 hinders DENV2 replication by the production of IFN-β4. TLR3 has also been shown to be upregulated in dengue virus-infected HepG2 cells [12]. Decreased DENV replication and increased anti-viral humoral response have been observed with the use of combined TLR 3/7/8 agonists in Macau [13]. A study conducted by Alagarasu et al. in dengue suggested that the T allele was also significantly more often found in cases as compared to controls in TLR3 rs3775291 gene polymorph [14]. Another study by Biyani et al. also investigated the association of the studied gene in Japanese encephalitis and a higher frequency of variant T allele was observed, similar to the present study [15]. TLR3 (rs3775291) is a well-studied polymorphism and has found to be associated with several other infectious diseases including severe tick-borne encephalitis (TBE) virus infection [16], human immunodeficiency virus (HIV) [ 17], and herpes simplex virus (HSV)-2 infections [18]. Despite a higher frequency of mutated alleles in cases, studied polymorphisms did not correlate with disease severity. Most dengue cases were asymptomatic or presented as classical dengue fever with only a few showing severe manifestations. The reason behind susceptibility to severe dengue is not clear. In the present study, heterozygous genotypes of TLR9 (rs187084, rs5743836) and of TLR7 (rs179008, rs179009) were found to be significantly associated with dengue cases as compared to controls. To the best of our knowledge, these polymorphisms of TLR7 and 9 have not been studied to date with dengue virus infection though TLR9 rs187084 & rs5743836 heterozygous genotype have been found to be significantly associated with neonatal severe hepatitis, neonatal Human Cytomegalovirus infection, malaria, pulmonary tuberculosis and autoimmune diseases [3]. Similarly, TLR7 rs179008 & rs5743836 are associated with cancer, malaria, HCV, and tuberculosis [6].

## Conclusions

The present study demonstrates that mutants of TLR3 rs3775291 and heterozygous genotypes of TLR7 and TLR9 are associated with susceptibility of dengue virus infection. Mutant alleles were present in increased numbers in rs3775290 and rs3775296 polymorphisms among dengue cases in comparison to controls but not at significant levels. Mutant allele of TLR7 and TLR9 was significantly associated with dengue.
